# Progress in acute myeloid leukaemia: small molecular inhibitors with small benefits

**DOI:** 10.3332/ecancer.2020.1015

**Published:** 2020-02-27

**Authors:** Talal Hilal

**Affiliations:** Division of Hematology/Oncology, University of Mississippi Medical Center, Jackson, MS, USA

**Keywords:** AML, glasdegib, gilteritinib, venetoclax, enasidenib, ivosidenib, clinical trials

## Abstract

The use of small molecule inhibitors in acute myeloid leukaemia (AML) has become ubiquitous with the US Food and Drug Administration approval of multiple agents between 2017 and 2018. Despite the promise, some of these indications are based on early efficacy data (phase I/II), and single-arm studies, and have not been tested in randomised trials. Furthermore, there are important limitations in the evidence that exists in randomised trials. This perspective aims to summarise the data that formed the basis for approval of gilteritinib, glasdegib, ivosidenib, enasidenib and venetoclax. It also aims to shed a light on some of the limitations in the evidence. Clinicians should exercise caution when using drugs that largely have yet to show an improvement in survival over the standard of care in AML.

Small molecule inhibitors are changing the landscape of therapy for many hematologic malignancies. This has been the most notable for chronic lymphocytic leukaemia and chronic myeloid leukaemia with agents, such as ibrutinib and imatinib, respectively. In the last few years, small molecule inhibitors have gained traction in the treatment of acute myeloid leukaemia (AML). Between August 2017 and November 2018, the US Food and Drug Administration (FDA) approved five small molecule inhibitors for the treatment of newly diagnosed and relapsed/refractory AML. The clinical benefit of these therapies is modest, but their uptake has been on the rise, with national guidelines, such as the National Comprehensive Cancer Network (NCCN), recommending them for indications that fall outside their tested purpose [1]. In this paper, the trials that led to the approval of this record number of AML therapies are reviewed and critiqued, ultimately attempting to answer the question, is the treatment landscape of AML changing for the better?

The cornerstone of treatment for patients with AML for the last 50 years has been intensive chemotherapy consisting of anthracycline plus cytarabine (so called 3+7 regimen) [2], which is associated with 70%–80% CR rate, and long-term survival, with consolidation high dose cytarabine or stem cell transplantation in high-risk patients, of about 50% [3]. The outcomes of AML in older patients (arbitrarily defined as >60 years in clinical trials) treated with a 3+7 regimen are inferior, with 50%–60% achieving CR that translate into a 2-year survival of about 15%–20% [4]. Furthermore, the majority of patients with AML are diagnosed at an older age and may have comorbidities that preclude the use of a 3+7 regimen. Treating AML in these older adults or those with comorbidities is therefore a therapeutic challenge [5]. Hypomethylating agents (HMAs) (e.g., decitabine and azacitadine) were introduced in the treatment landscape and have been shown to improve outcomes of older patients with AML, and are considered standard of care treatments for patients ineligible for intensive chemotherapy [6].

The recent FDA approvals of glasdegib [7], gilteritinib [8], venetoclax [9, 10], enasidenib [11] and ivosidenib [12] have challenged this paradigm. These five small molecule inhibitors have various targets including the Hedgehog pathway, FMS-related tyrosine kinase 3 (FLT3), B-cell lymphoma-2 (BCL2) antiapoptotic protein and isocitrate dehydrogenase 1 and 2 (IDH 1 and 2). Details of each trial that served as the basis for approval of these agents are listed in Table 1.

Enasidenib, an IDH2 inhibitor, was the first to be approved on the basis of a phase Ib/II trial that assessed safety, and clinical activity in patients with IDH2-mutated advanced myeloid malignancies [11]. In the dose-expansion phase, the 100 mg dose of enasidenib was administered to 109 patients with relapsed/refractory disease. The overall response rate (ORR) was 38.5%, with a complete remission (CR)/complete remission with incomplete hematologic recovery (CRh) rate of 26.6%. Importantly, median duration of response (DOR) was 5.6 months (95% CI 3.8–9.7), and median OS was 9.3 months (95% CI 8.2–10.9).

The study population included patients who were refractory to initial induction or re-induction (32%), patients who had relapsed disease within 1 year of therapy (25%), and patients who had relapsed and did not respond to two or more cycles of first-line lower-intensity therapy (e.g., decitabine, azacitadine) (23%). This is problematic because in at least 23% of patients, lower-intensity therapy was perhaps prematurely discontinued after two cycles, despite median time to best response taking approximately 90 days in seminal trials of hypomethylating agents versus conventional care [6, 13]. Furthermore, in 25% of patients who relapsed within 1 year of therapy, second-line therapy was not offered, which is typically associated with a CR/CRh rate of 50%–60% if treated with aggressive therapy with intent for transplant [14, 15].

Ivosidenib, an IDH1 inhibitor, was approved on the basis of similar quality evidence to enasidenib. A first-in-class human phase Ib/II trial in patients with IDH1-mutated relapsed or refractory AML showed a CR/CRh rate of 30%, DOR of 8.2 months (95% CI 5.5–12), and median OS of 8.8 months (95% CI 6.7–10.2) [12]. The study population included more patients refractory to initial induction or reinduction therapy (69%). However, it was unclear how many patients received reinduction, and what regimens were administered. Ivosidenib received regular approval, and the FDA did not require post-approval randomised trials.

Several studies evaluating the outcomes of patients with IDH1/2 mutations have reported conflicting results on the prognostic relevance of these mutations. In one of the largest studies, the presence of IDH 1/2 mutations did not seem to influence ORR or CR/CRh rates compared with IDH wild-type patients. Patients with IDH 1/2 relapsed AML treated with salvage therapy had a median OS of 11.1 months and 5.9 months, respectively [16]. These survival rates are not drastically different from the phase I/II trials discussed earlier [12]. A comparison between enasidenib or ivosidenib to standard salvage therapies or HMAs may have yielded unexpected results.

Venetoclax, a BCL-2 inhibitor, was approved on the basis of two phase Ib/II trials in patients with previously untreated AML who are 75 years or older or who have comorbidities that preclude the use of intensive induction chemotherapy. Efficacy was established based on the CR/CRh rate and the duration of CR. The first trial evaluated venetoclax with HMAs and reported a CR/CRh rate of 67% with a median duration of CR/CRh of 11.3 months (95% CI, 8.9—NR) [9]. The second evaluated venetoclax with low-dose cytarabine (LDAC) and reported a CR/CRh rate of 54% with a median duration of CR/CRh of 8.1 months (95% CI, 5.3–14.9 months) [10]. Patients over the age of 75 years comprised the minority of participants in the key registration trial of venetoclax plus azacitadine or decitabine (30%), the majority had an ECOG performance status of 0–1 (80%–85%), and none had a performance status of 3 or 4. These are patients that would likely have been offered intensive chemotherapy in the real-world setting. Older patients (75 years or older) with an ECOG performance status of 0–2 were reported to have 3-year survival rates of 30% in the German AML Cooperative Group trial [17]. Furthermore, it was unclear how many were unfit for intensive therapy based on comorbidities or the effects of those comorbidities on outcomes.

Glasdegib, a Hedghog pathway inhibitor, was approved on the basis of BRIGHT AML 1003, an open-label, multi-center phase II trial, which compared glasdegib (with LDAC) to LDAC alone for older patients (>75 years) or those with comorbidities with newly diagnosed AML. BRIGHT AML 1003 showed an improved OS with the glasdegib combination [median OS of 8.8 months versus 4.9 months (95% CI 0.39–0.67)] [18]. Importantly, event-free survival (EFS) data were not reported in the manuscript, which are essential to better evaluate the investigational agent since OS can be influenced by subsequent therapies after progression of disease on the investigational agent. Similarly to the venetoclax trials, the BRIGHT AML 1003 trial used age and comorbidities as surrogate markers for fitness, and included 40% of patients who were below the age of 75 years, and 50% with an ECOG performance status of 0–1. The determination that these patients as unfit for intensive chemotherapy is notoriously subjective [19]. Better tools for assessment of eligibility for intensive induction in older patients, including online tools for predicting treatment-related mortality and geriatric assessments, which are more predictive of outcomes compared to age and/or selected comorbidities would have been more reliable [19, 20].

Another important criticism of this trial is its use of an agent that is suboptimal and not commonly utilised in the USA. HMAs are preferred low-intensity therapies based on 2 phase III trials. The first showed superior OS with azacitadine compared to a conventional care regimen (including LDAC, intensive chemotherapy or supportive care) in patients with low marrow blast count AML [21]. The second showed superior response rate of decitabine in older patients >65 years with AML compared to treatment choice (LDAC or supportive care). A post-hoc analysis showed a survival advantage of decitabine [22]. In fact, 40% of patients randomised to the investigational arm of BRIGHT AML 1003 received subsequent chemotherapy with azacitadine and decitabine after progression, compared to 34% on the control arm.

Gilteritinib, a FLT3 inhibitor, was approved in November 2018 for relapsed/refectory AML with FLT3 mutation on basis of an interim analysis of the phase 3 ADMIRAL trial that reported a CR/CRh rate of 21% (95% CI: 14.5, 28.8) [23]. Updated results of the trial showed improved OS with gilteritinib versus standard of care (i.e., mitoxantrone/etoposide/cytarabine, fludarabine/cytarabine/granulocyte colony-stimulating factor/idarubicin, LDAC and azacitadine), with a median OS of 9.3 months versus 5.6 months [hazard ratio (HR) for death = 0.637; *p* = 0.0007]; 1-year survival rates were 37.1% and 16.7%, respectively [8, 24]. The trial had several points in its favour, including allowing prior frontline use of midostaurin or sorafenib before randomisation, and having multiple choices of standard therapies in the control arm. However, the proportion of patients who received low-intensity therapy (i.e., LDAC or azacitadine) is substantial (40%), and the reason salvage chemotherapy with a higher intensity regimen (which is expected to yield a higher RR) was not offered to those patients is unclear. This may have skewed results in favour of gilteritinib if efficacy was compared to lower-intensity regimens that are associated with lower response rates when administered to patients who are otherwise candidates for high-intensity chemotherapy.

In a malignancy that is traditionally curable with high-intensity chemotherapy and allogeneic stem cell transplantation, and where outcomes of patients with relapsed/refractory disease are poor, novel agents with superior efficacy to standard of care are desperately needed. However, as shown in this paper, the approvals of enasidenib, ivosidenib and venetoclax are currently based on single-arm trials, and their superiority over standard of care therapy remains to be seen. Glasdegib has not been compared to the most commonly used standard of care (i.e., decitabine and azacitadine), and EFS data are not reported. Gilteritinib may have fared differently if compared to intensive chemotherapy.

Furthermore, in the case of glasdegib, an approval in the front-line setting for an agent to be added to (not replace) the existing standard of care, it is important to demonstrate improved long-term survival to justify the additional burden—physical, logistic, financial and others—of added therapy. For all five of these agents, efficacy endpoints that include health-related quality of life (HRQOL) outcomes, compared to control arm or baseline, would have strengthened the argument in favour of these agents. As discussed above, the improvement in median survival is approximately 3–4 months [7, 8]. A remission that lasts a few months longer can be outweighed by the increased toxicity of these drugs.

Although the FDA has an accelerated approval path to grant access to drugs that treat serious conditions, and that fill an unmet medical need based on a surrogate endpoint, it is somewhat surprising to see that four of these approvals were ‘regular’ approvals, venetoclax being the only agent receiving accelerated approval. Accelerated approvals require the manufacturer to conduct a postmarketing study confirming meaningful benefit of the drug while regular approval may not always require such commitments. Moreover, key phase III trials currently ongoing may fail to answer such clinically important questions. For example, ivosidenib versus placebo in combination with azacitadine in previously untreated patients (NCT03173248) will fail to answer the question of whether intensive chemotherapy added to ivosidenib would be more efficacious than intensive chemotherapy alone. Venetoclax in combination with azacitadine versus azacitadine in treatment naïve AML (NCT02993523) will fail to answer the question of whether venetoclax with azacitadine is superior to intensive chemotherapy alone.

## Conclusion

It is encouraging to see novel therapies make their way in the treatment landscape of AML. However, in at least four of the five recent approvals, efficacy over standard of care therapies remains questionable largely due to design flaws in the trials including the use of suboptimal control arms, as well as their nonrandomised nature. National guidelines (e.g., NCCN) should avoid encouraging the use of some of these agents outside their approved indications (e.g., ivosidenib or enasidenib for frontline therapy), which may lead to increased uptake by clinicians without proven benefit over alternatives. A focus on HRQOL outcomes, especially in the relapsed/refractory setting, should be of priority in future trials. In the absence of more definitive data, clinicians should be cognizant of the limitations in the currently available evidence.

## Conflicts of interests

None declared.

## Funding

No source of funding was used in the writing of this article.

## Figures and Tables

**Table 1.** Trials that led to FDA approval of small molecule inhibitors in AML.

Registration Trial	Trial Type	Indication	Investigational Arm	Control Arm	Sample Size	Primary Endpoint	Median Follow-up (months)	Response				Overall Survival	
								CR/CRh	Duration of CR/CRh (months)	80/95% CI	1 year OS	Median (months)	80/95% CI (months)
ADMIRAL	Phase III	FLT3+, relapsed	Gilteritinib	Salvage chemotherapy	138	OS and CR/CRh	4.6	34% (versus 15%)	NR	14.5 to 28.8	37% (versus 17%)	9.3 (versus 5.6)	7.7 to 10.7
BRIGHT AML 1003	Phase II	≥ 75 years, or ≥ 55 years with comorbidities	Glasdegib (with LDAC)	LDAC	115	OS	21.7	CR only: 17% (versus 3%)	9.9	12.4 to 24.8	39.5%	8.8 (versus 4.9)	6.9 to 9.9
Study M14-358	Phase Ib/II	≥ 75 years, untreated AML, or ≥ 65 ineligible for intensive chemotherapy	Venetoclax (with azacitadine or decitabine)	None	145	Safety, CR/CRh	15.1	73%	12.5	11 to NR	59%	17.5	12 to NR
Study M14-387	Phase Ib/II	≥ 70 years, untreated AML, or ≥ 60 ineligible for intensive chemotherapy	Venetoclax (with LDAC)	None	82	Safety, CR/CRh	NR	54%	8.1	5.3 to 14.9	40%	10.1	5.7 to 14.2
AG221-C-001	Phase Ib/II	Relapsed/ refractory with IDH2 mutation	Enasidenib	None	199	Safety, CR/CRh	7.7	23%	8.8	6.4 to NR	39%	9.3	8.2 to 10.9
AG120-C-001	Phase Ib/II	Relapsed/ refractory with IDH1 mutation	Ivosidenib	None	174	Safety, CR/CRh	14.8	30%	8.2	5.5 to 12	34%	8.8	6.7 to 10.2
